# Identification of potential genes involved in triterpenoid saponins biosynthesis in *Gleditsia sinensis* by transcriptome and metabolome analyses

**DOI:** 10.1007/s11418-018-1270-2

**Published:** 2018-12-13

**Authors:** Yusuke Kuwahara, Daisuke Nakajima, Sayaka Shinpo, Michimi Nakamura, Noriaki Kawano, Nobuo Kawahara, Mami Yamazaki, Kazuki Saito, Hideyuki Suzuki, Hideki Hirakawa

**Affiliations:** 1 Graduate School of Life Sciences, Tohoku University, Aoba-ku, Sendai, Miyagi 980-8577 Japan; 2 Kazusa DNA Research Institute, 2-6-7 Kazusa-kamatari, Kisarazu, Chiba 292-0818 Japan; 3 Graduate School of Pharmaceutical Sciences, Chiba University, 1-8-1 Inohana, Chuo-ku, Chiba, 260-8675 Japan; 4 Tsukuba Division, Research Center for Medicinal Plant Resources, National Institutes of Biomedical Innovation, Health and Nutrition, Hachimandai 1-2, Tsukuba-shi, Ibaraki 305-0843 Japan

**Keywords:** Saponin biosynthesis, De novo transcriptome assembly, *Gleditsia sinensis*, P450, UGT

## Abstract

*Gleditsia sinensis* is widely used as a medicinal plant in Asia, especially in China. Triterpenes, alkaloids, and sterols were isolated from *Gleditsia* species. Among them, triterpenoid saponins are very important metabolites owing to their various pharmacological activities. However, the triterpenoid saponin biosynthesis pathway has not been well characterized. In the present study, we performed de novo transcriptome assembly for 14.3 Gbps of clean reads sequenced from nine tissues of *G. sinensis*. The results showed that 81,511 unique transcripts (unitranscripts) (47,855 unigenes) were constructed, of which 31,717 unigenes were annotated with Gene Ontology and EC numbers by Blast2GO against the NCBI-nr protein database. We also analyzed the metabolite contents in the same nine tissues by LS–MS/MS, and saponins including gleditsioside I were found in fruit at higher levels. Many of the genes with tissue-specific expression in fruit are involved in the flavonoid biosynthesis pathway, and many of those have UDP-glucosyltransferase (UGT) activity. We constructed a saponin biosynthesis pathway and identified two key enzyme families in the triterpenoid saponin biosynthesis pathway, cytochrome P450 and UDP-glucosyltransferase, that are encoded by 37 unigenes and 77 unigenes, respectively. CYP72A, CYP716A, and CYP88D, which are known as key enzymes for saponin biosynthesis, were also identified among the P450s. Our results provide insight into the secondary metabolite biosynthesis and serve as important resources for future research and cultivation of *G. sinensis*.

**Electronic supplementary material:**

The online version of this article (10.1007/s11418-018-1270-2) contains supplementary material, which is available to authorized users.

## Introduction

*Gleditsia* is a genus from the legume family (Fabaceae) and grown worldwide, especially in Asia and America. Many species in the *Gleditsia* genus have been identified, including *G. sinensis*, *G. japonica*, and *G. triacanthos*, and these and other *Gleditsia* species are used as traditional diuretics and expectorants [1]. Recent phytochemical studies have revealed the presence of triterpenoid saponins, alkaloids, sterols, and flavonoids in these species. The extracts of these compounds exhibit several important biological activities, including antitumor, anti-inflammatory, and anti-HIV activities [2–4]. Over 30 compounds of triterpenoid saponins were identified in the tissues of *G. sinensis* [1], but the biosynthesis of these physiologically active substances is not yet fully understood.

Along with advances in the technology of DNA sequencers, the genomic and transcriptomic information of medicinal plants, e.g., *Catharanthus roseus, Glycyrrhiza uralensis*, and *Panax notoginseng*, has been accumulating [5–7]. Large international consortiums such as the 1000 Plant Project have specifically targeted medicinal plants [8]. RNA-sequencing (RNA-Seq) technology is one of the powerful approaches for identifying functional genes without genome sequences. Two RNA-Seq studies have been conducted in *G. sinensis*. Zhu et al. analyzed the de novo transcriptomes from four tissues [9] and Han et al. reanalyzed the same transcriptome data set and identified putative chalcone isomerase genes [10]. However, these studies were not sufficient to understand differences in the bioactive metabolite biosynthesis pathway among a variety of tissues and to identify key enzymes for bioactive metabolites including saponins. Therefore, in this study, we conducted de novo assembly for the transcriptome data obtained from the nine tissues of *G. sinensis* and obtained annotated unigenes. This result could provide useful information for the mining of functional genes for the stable production of bioactive compounds of *G. sinensis*.

## Materials and methods

### Plant materials, RNA isolation, and library preparation

**Fig. 1:**
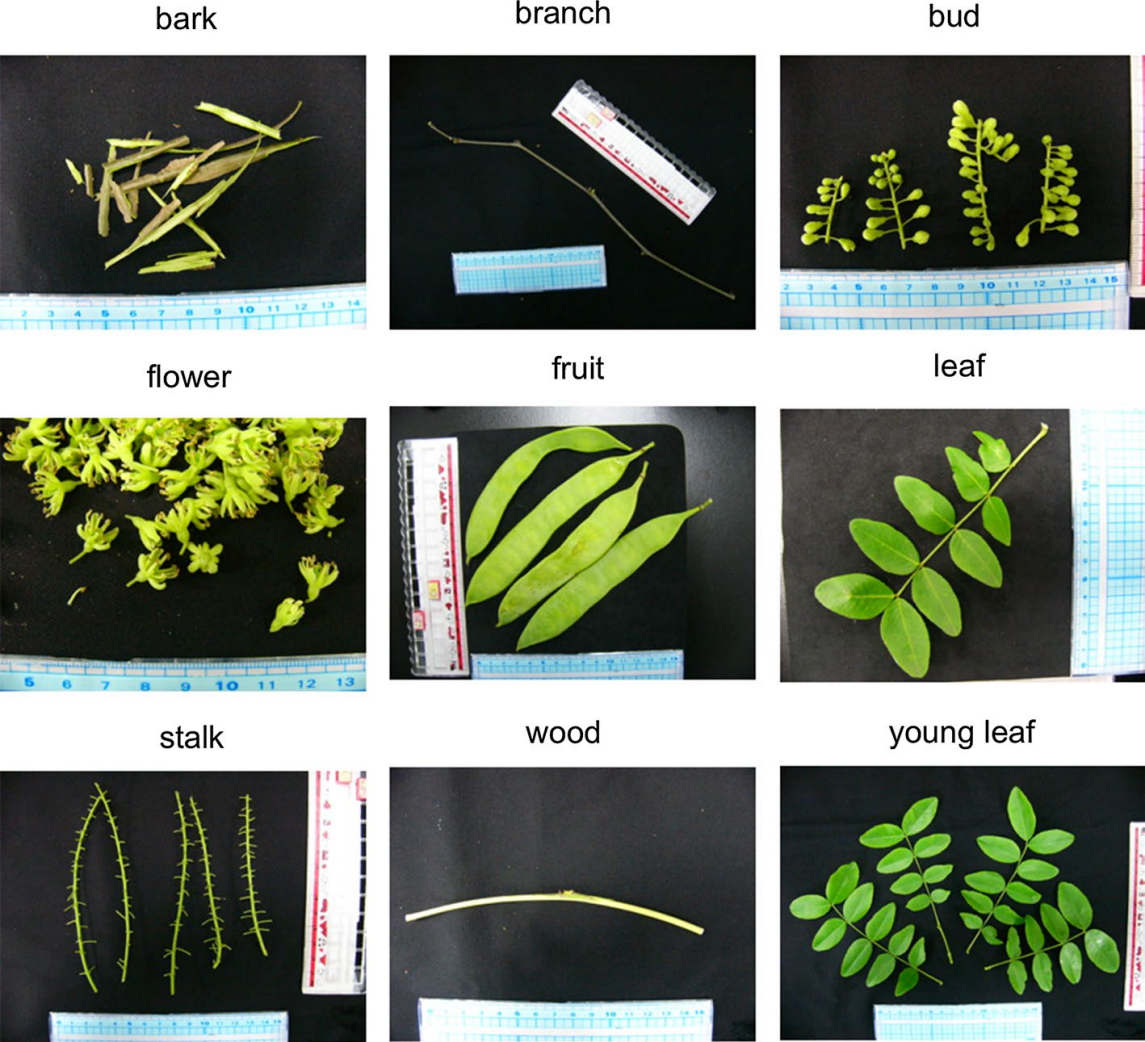
Nine tissues of *Gleditsia sinensis* used for de novo transcriptome analysis. RNA extraction and RNA-sequencing were conducted against the nine tissues (bark, branch, bud, flower, fruit, leaf, stalk, wood, and young leaf)

The nine tissues examined were bark, branch, bud, flower, fruit, leaf, stalk, wood, and young leaf; all were harvested from natural growing plants between May and June 2013 at Inohana Campus, Chiba University, Japan (Fig. 1). All tissues were frozen with liquid nitrogen until total RNA extraction.

### Illumina sequencing

Total RNA extraction was performed using an RNeasy Mini kit (Qiagen, Valencia, CA) and the qualities were assessed by using a Bioanalyzer 2100 (Agilent, Palo Alto, CA). The samples with an RNA integrity number (RIN) above 7.8 were used for further analysis. The cDNA library was constructed using an Illumina TruSeq Prep kit v2 according to the manufacturer’s protocol (Illumina, San Diego, CA). The resulting cDNA library was sequenced using a HiSeq 1500 system (Illumina) with 100 bp paired-end reads.

### De novo transcriptome assembly

The quality of the raw reads was checked by FastQC [11] and then the reads were processed by Trimmomatic software version 0.36 [12] to remove adaptor sequences and unpaired reads. Reads with a sequence length less than 75 bp were also removed. We performed de novo assembly using Trinity version 2.3.2 [13] with cleaned reads from all the samples to construct the unique transcripts (contigs). To determine the gene expression levels, we used RSEM [14] and filtered out the transcripts expressed at low levels with fragments per kilobase of transcript per million mapped reads (FPKM) < 1. The longest isoforms within the remaining transcripts were selected as unigenes and used for further analyses.

### Functional annotation

All unigenes were searched against the NCBI-nr protein database (formatted in May, 2017) with Blastx (*E* value cutoff of 1e−5) and annotated by Blast2GO version 4.1.9 [15] with default settings. The GO categories, EC numbers, and gene IDs of the KEGG pathways [16] were also assigned and the top 20 gene ontology terms from three categories (biological process, molecular function, and cellular component) at level 3 for unigenes and the species distribution of the top Blastx hits were visualized by Blast2GO.

### Expression analysis

The expression levels based on counts per million (CPM) in each sample were calculated against the unigenes by Bowtie2 in RSEM. The raw counts were normalized by trimmed-mean of M normalization (TMM) and transformed to CPM with EdgeR for further analysis [17]. Principal component analysis was conducted with R.

### GO enrichment analysis of tissue-specific gene expression

We identified the genes specifically expressed in each of the nine tissues using the tissue specificity index tau, which ranged from 0 (expressed in all tissues) to 1 (expressed in a single tissue) [18]. Tau was calculated on TMM-normalized CPM and the threshold was set to 0.95 for the tissue-specific genes. GO enrichment analysis was performed with the goseq [19] bioconductor package using hypergeometric testing and the overrepresented *p* value cutoff of < 0.01.

### Selection of candidate genes associated with the saponin biosynthesis pathway

The putative saponin biosynthesis pathway was constructed on the basis of the terpenoid backbone pathway (map00900) in KEGG, and the enzymes were mapped on the basis of the EC numbers annotated by Blast2GO. The putative open reading frames (ORFs) were obtained by Blast2GO on the basis of the top hit, and those less than 100 amino acid residues in length were excluded. The predicted protein sequences were searched against the Pfam database by HMMER version 3.1b2 (*E* value cutoff of 1e−5). Candidate P450s and UGTs were predicted if the unigenes were assigned to PF00067 (cytochrome P450) and PF00201 (UDP-glucuronosyl and UDP-glucosyl transferase), respectively. To select the candidate P450 involved in saponin biosynthesis, all P450s were searched against predicted P450s in three Fabaceae species (*Lotus japonicus*, *Cicer arietinum*, and *Cajanus cajanifolius*) from the cytochrome P450 homepage (https://drnelson.uthsc.edu/CytochromeP450.html) by Blastp (*E* value cutoff of 1e−5 and percentage identity cutoff of 40%).

### Metabolite extraction

Each tissue of *G. sinensis* was immediately frozen in liquid nitrogen and ground to powder using a mortar and pestle. Powdered samples (100 mg) were extracted with three volumes of methanol. After two homogenizations using a TissueLyser (Qiagen) at 27 Hz for 2 min each, the homogenates were centrifuged (12,000 × *g*, 10 min, 4 °C). The supernatant was filtered through a C18 Spin column (GL Sciences, Tokyo), and the filtrate was used for LC-Orbitrap-MS analysis.

### HPLC–MS analytical conditions

HPLC–MS was performed with an Agilent 1200 system (Agilent) coupled to a Finnigan LTQ Orbitrap XL (Thermo Fisher Scientific, Waltham, MA), which was equipped with an electrospray source operating in the negative-ionization mode. An aliquot of the extracted sample (5 μL) was injected into an Ascentis Express C18 (2.7 µm, 150 × 4.6 mm; Sigma, St. Louis, MO) with mobile phases that consisted of 0.1% (v/v) aqueous formic acid (solvent A) and 0.1% (v/v) formic acid in acetonitrile (solvent B). The gradient program was as follows: 30–60% solvent B for the first 48 min, 60–95% solvent B for the next 2 min, 95% solvent B for the next 5 min, and 30% solvent B for the last 5 min, with a flow rate of 0.5 mL/min. The column oven temperature was set at 40 °C. HPLC–MS analysis was performed using electrospray ionization (ESI) in negative-ionization mode. The full scan (*m*/*z* 800–2000) used a resolution of 60,000. MS/MS were acquired on a Top5 data-dependent mode with a parent list for saponins.

### Data analysis of HPLC–MS

All data obtained from the HPLC–MS analysis were acquired with the Xcalibur™ software (Thermo Fisher Scientific). Detection and structural prediction of saponins were determined by comparing the obtained data with the accurate mass, MS/MS spectra, and retention time from the previous data set (Supplementary Table S3) [20]. Full MS data and MS^2^ analysis for gleditsioside I are shown in Supplementary Fig. 1.

## Results

### RNA-sequencing and de novo transcriptome assembly

A total of 14.3 Gbp reads from the nine tissues of *G. sinensis* were checked for quality and then applied to the transcriptome analysis. About 95% of the raw reads were of good quality (quality value (QV) > 30). The high-quality cleaned reads were used for de novo transcriptome assembly with Trinity. We obtained 230,780 contigs and estimated the expression levels with Bowtie2 in RSEM. The 81,511 contigs were selected as unique transcripts (unitranscripts) after removing the genes with low gene expression of FPKM < 1. Moreover, the longest isoforms were selected from each of the unitranscripts as unigenes, because the splicing variants were included in the unitranscripts. As a result, the average, N50, and maximum lengths of the 47,855 unigenes were 1103 bases, 1952 bases, and 17,250 bases, respectively, and their GC content was 40.7% (Table 1).

**Table 1:** Summary of de novo transcriptome assembly

Read processing	
Total cleaned reads	143,593,236
Total length of cleaned reads (bp)	14,318,906,260
Trinity de novo assembly	
Number of contigs	230,780
Total length of contigs (bp)	240,698,197
Average length of contigs (bp)	1043
Maximum length of contigs (bp)	17,250
Minimum length of contigs (bp)	201
Contig N50 (bp)	1985
Contig GC (%)	40.5
Number of unitranscripts	81,511
Total length of unitranscripts (bp)	107,461,949
Average length of unitranscripts (bp)	1318
Maximum length of unitranscripts (bp)	17,250
Minimum length of unitranscripts (bp)	201
Unitranscript N50 (bp)	2064
Unitranscript GC (%)	41.0
Number of unigenes	47,855
Total length of unigenes (bp)	52,773,871
Average length of unigenes (bp)	1103
Maximum length of unigenes (bp)	17,250
Minimum length of unigenes (bp)	201
Unigene N50 (bp)	1952
Unigene GC (%)	40.7

### Functional annotation

**Fig. 2:**
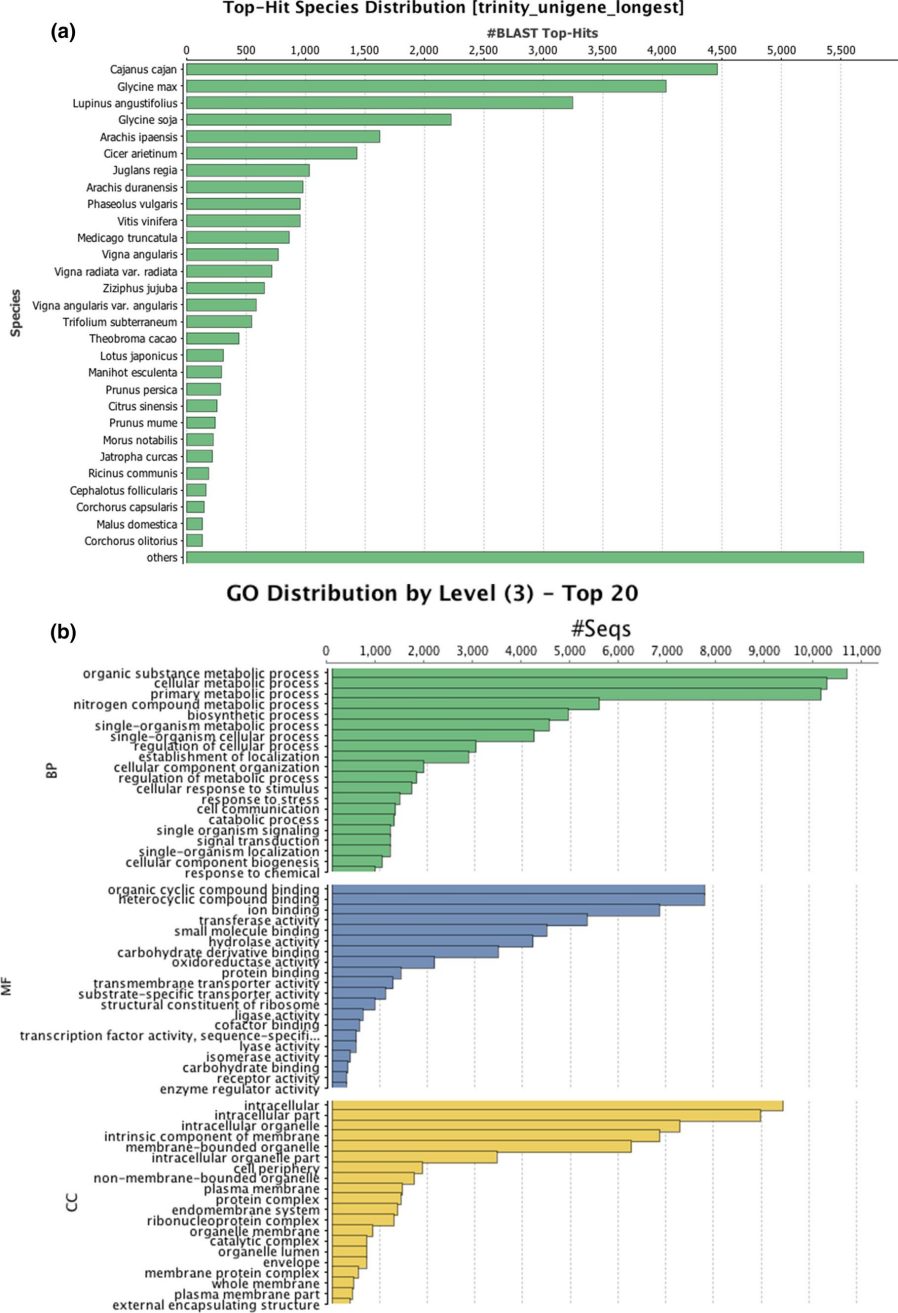
**a** Species distribution of the unigenes based on Blast2GO analysis. The species of the unigenes were estimated by the top hits against the NCBI-nr protein database using Blastx in Blast2GO. **b** Gene ontology (GO) distributions for the unigenes. The top 20 GO terms at level 3 based on the number of assigned unigenes for biological process (BP), molecular function (MF), and cellular component (CC) were investigated using Blast2GO

The Blast2GO searches successfully annotated the functions of 31,717 of the 47,855 unigenes (66.3%). The distribution of the species of the entries is shown in Fig. 2a, and 80% of the annotated unigenes were highly similar to genes of *Fabaceae* family members (*Cajanus cajan*, *Glycine max*, *Lupinus angustifolius*, and *Glycine soja*). The top 20 annotated gene ontology terms at the level 3 annotation with three categories (biological process, molecular function, and cellular component) are shown in Fig. 2b. The top five GO biological process terms were involved in metabolic processes (organic substance metabolic process (GO: 0071704), cellular metabolic process (GO: 0044237), primary metabolic process (GO: 0044238), nitrogen compound metabolic process (GO: 0006807), and biosynthetic process (GO: 0009058). Organic cyclic compound binding (GO: 0097159), heterocyclic compound binding (GO: 1901363), ion binding (GO: 0043167), transferase activity (GO: 0016740), and small molecule binding (GO: 0036094) were the top five GO molecular function terms. Intracellular (GO: 0005622), intracellular part (GO: 0044424), intracellular organelle (GO: 0043229), intrinsic component of membrane (GO: 0031224), and membrane-bounded organelle (GO: 0043227) were the top five GO cellular component terms.

### Expression analysis

**Fig. 3:**
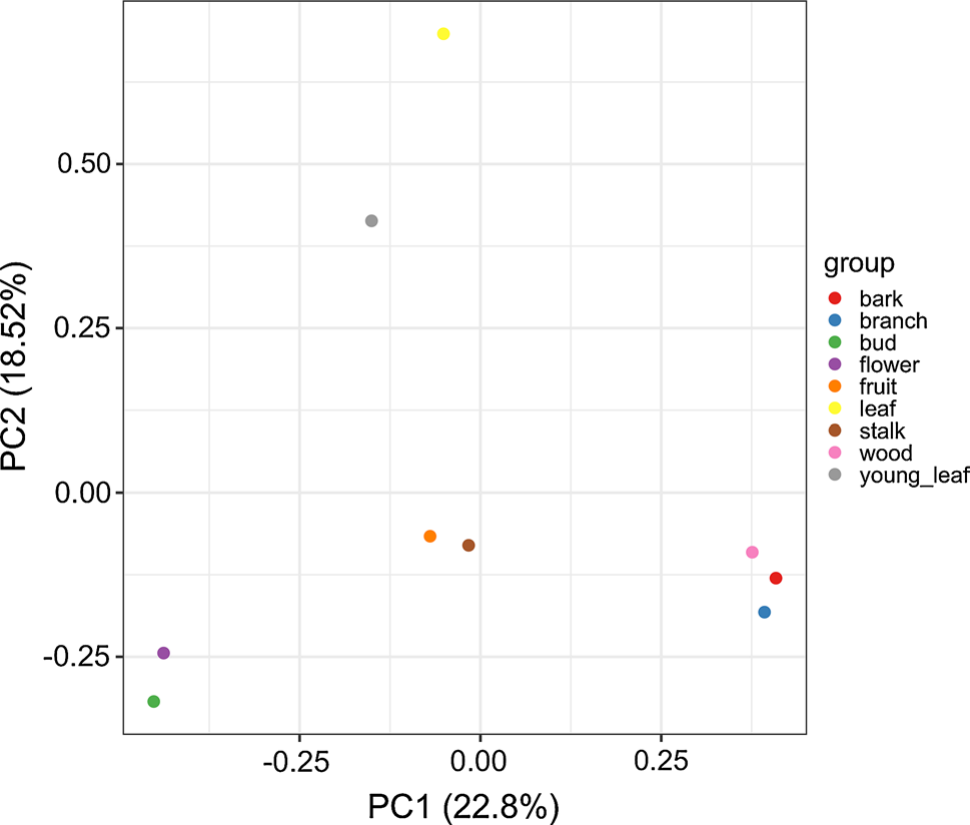
Principal component analysis for the expression of the unigenes among the nine tissues (color figure online)

To estimate the abundance of expression of genes in the nine tissues, all the cleaned reads were mapped to the contigs constructed by de novo transcriptome assembly using Bowtie2 in RSEM. The raw count was normalized between tissues using the trimmed-mean of M normalization (TMM) method and transformed to counts per million (CPM) using edgeR. In order to compare the gene expression patterns among the nine tissues, we performed principal component analysis (PCA) using the prcomp function in R. PCA revealed that the nine tissues were clustered along developmental organs (Fig. 3). Two major components accounted for 41% of the gene expression variance. Along the PC2 axis, the results showed that gene expressions in the leaf and young leaf (yellow and gray) were significantly different from those in the other tissues. Along the PC1 axis, the other tissues were clustered into three new groups: branch, wood, and bark (blue, pink, and red); fruit and stalk (orange and brown); and bud and flower (green and purple). These results showed that the gene expression dynamics differed among the nine tissues.

**Fig. 4:**
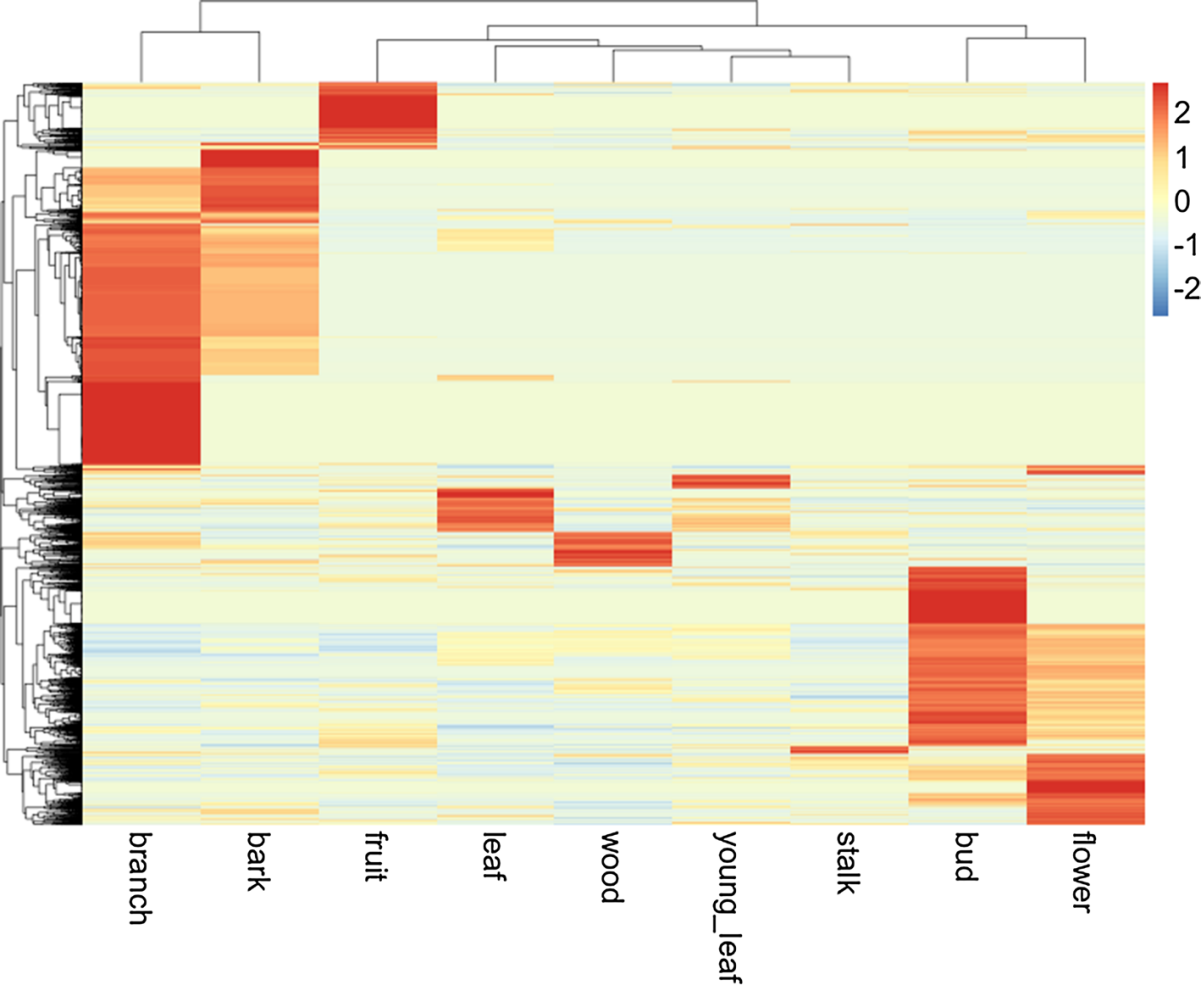
Hierarchical clustering of tissue-specific unigenes in the nine tissues. The color key represents normalized log2 (CPM) values (color figure online)

**Fig. 5:**
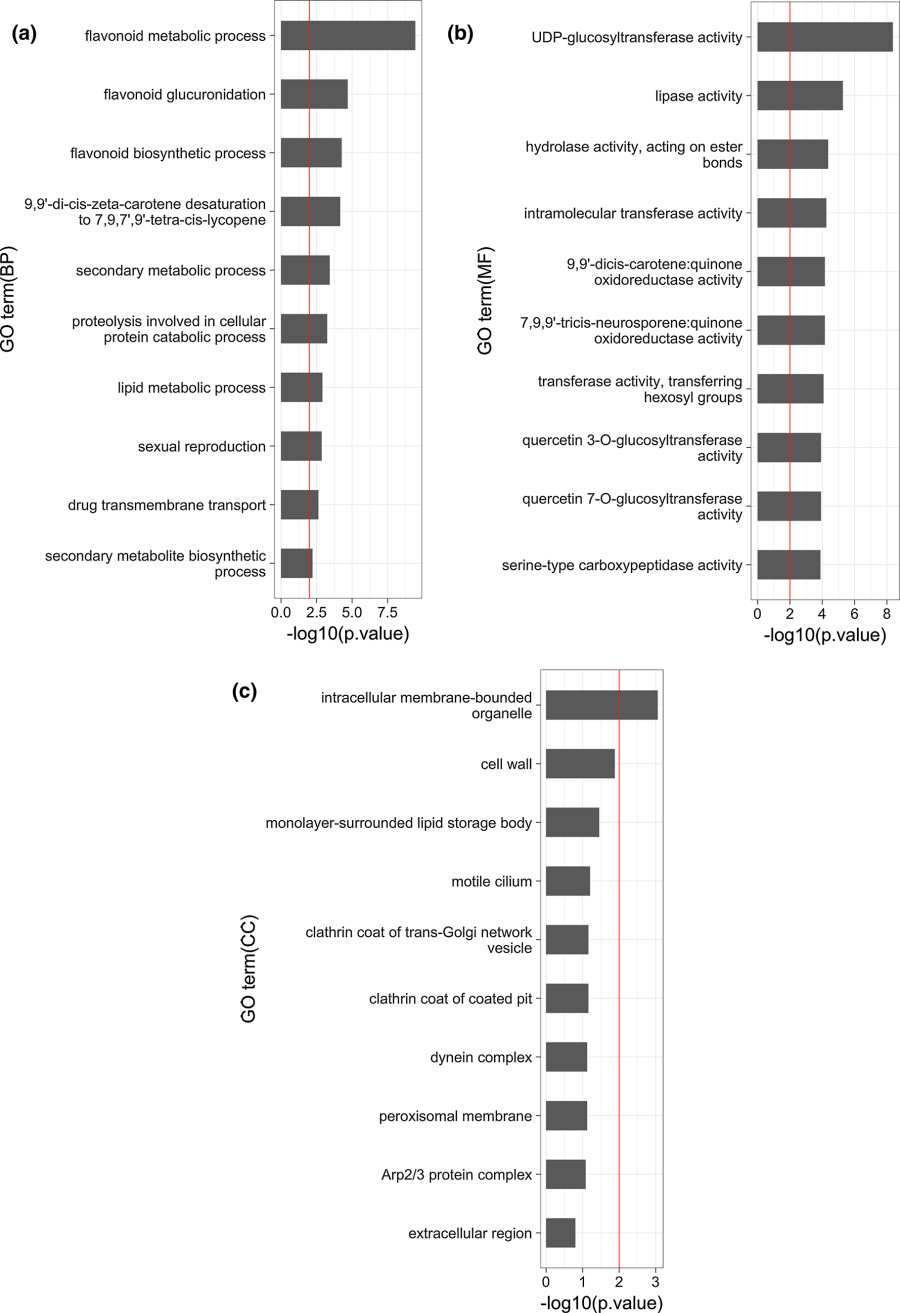
GO enrichment analysis for the unigenes specifically expressed in fruit. GO enrichment analysis using GOseq for 341 fruit-specific unigenes with tau > 0.95. The red line indicates statistical significance (*p *value = 0.01). Biological process (BP, **a**), molecular function (MF, **b**), and cellular component (CC, **c**) (color figure online)

Next, we analyzed the tissue specificity of the highly expressed genes in each of the nine tissues based on the tissue specificity index (tau). The tau value ranged from 0 to 1, where 1 means that a gene is highly expressed in only one tissue [18]. We identified 3959 genes (8.2%) with tau > 0.95 as tissue-specific genes. A heatmap displayed differential expression patterns of the tissue specific genes in the nine tissues: 77 genes in young leaf, 228 genes in leaf, 1335 genes in branch, 180 genes in wood, 381 genes in bark, 955 genes in bud, 421 genes in flower, 41 genes in stalk and 341 genes in fruit (Fig. 4). Many branch-specific genes were expressed in bark, and many of bud-specific genes were expressed in flower. On the other hand, the fruit-specific genes were not highly expressed in the other tissues. In order to clarify the different biological functions associated with the different tissue-specific genes, we performed GO enrichment analysis. The top 10 GO terms in fruit-specific genes are shown in Figs. 5a–c. Figure 5a shows that five ontology terms were significantly enriched in biological process (BP). Top three enriched terms were flavonoid metabolic process (GO: 0009812, *p *value = 3.58e−10), flavonoid glucuronidation (GO: 0052696, *p *value = 2.02e−5), and flavonoid biosynthetic process (GO: 0009813, *p *value = 5.37e−5). In the molecular function (MF) terms, Fig. 5b shows that 12 GO terms were significantly enriched with *p *values < 0.01 and top three enriched terms were UDP-glycosyltransferase activity (GO: 0035251, *p *value = 4.26e−9), lipase activity (GO: 0016298, *p *value = 5.42e−6), and hydrolase activity acting on ester bonds (GO: 0016788, *p *value = 4.34e−5). Figure 5c shows that intercellular membrane-bounded organelle (GO: 0043231, *p *value = 8.84e−4) was the most significantly enriched term in cellular component (CC). All significant GO terms in the other tissues are presented in Supplementary Table S1.

### Saponin analysis

**Fig. 6:**
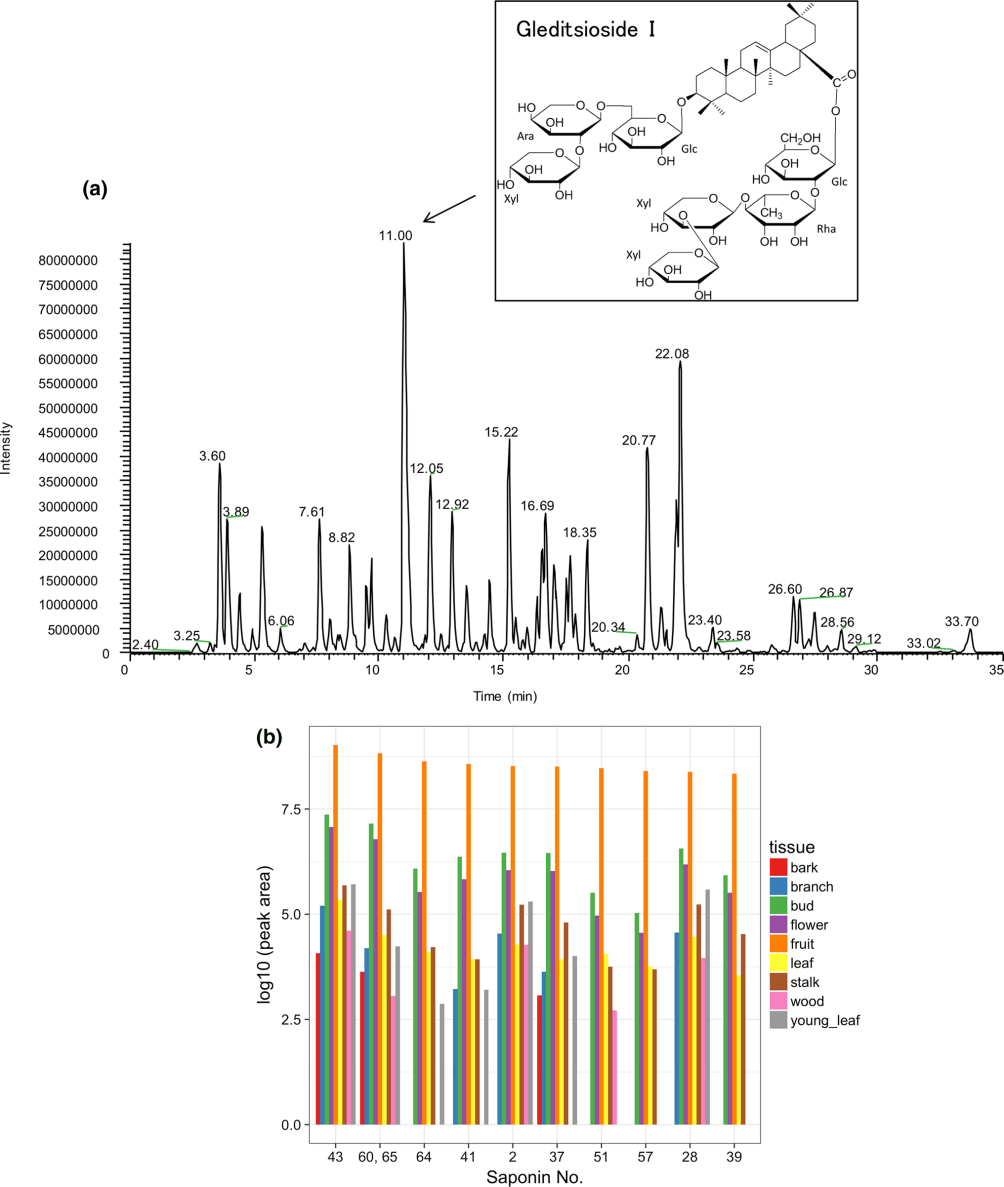
Metabolomic analysis for the nine tissues by LC/MS. Base peak chromatogram of a fruit extract of *G. sinensis* by LS/MS (**a**) and the content of the top 10 saponins in fruit (**b**). The elution peak (Rt =11.00 min, *m/z* = 1453.6849) detected and speculated as saponin of gleditsioside I which is compared to data of compound no. 0.43 of Wang et al. [20]. Glc: glucose, Ara: arabinose, Rha: rhamnose, Xyl: xylose. The saponin nos. are described in Supplementary Table S3 in detail

We analyzed the content of saponins among the nine tissues using LC–MS/MS. The base peaks of the metabolite extracted from fruits are shown in Fig. 6a. Gleditsioside I was the most highly detected saponin in the fruit of *G. sinensis*. Gleditsioside I was previously isolated from fruit [21], which was consistent with our result. The profiles in each tissue of the top 10 saponins with the highest contents in fruit are shown in Fig. 6b. Almost all the saponins were detected at higher levels in fruit, bud, and flower.

### Identification of genes involved in saponin biosynthesis

**Fig. 7:**
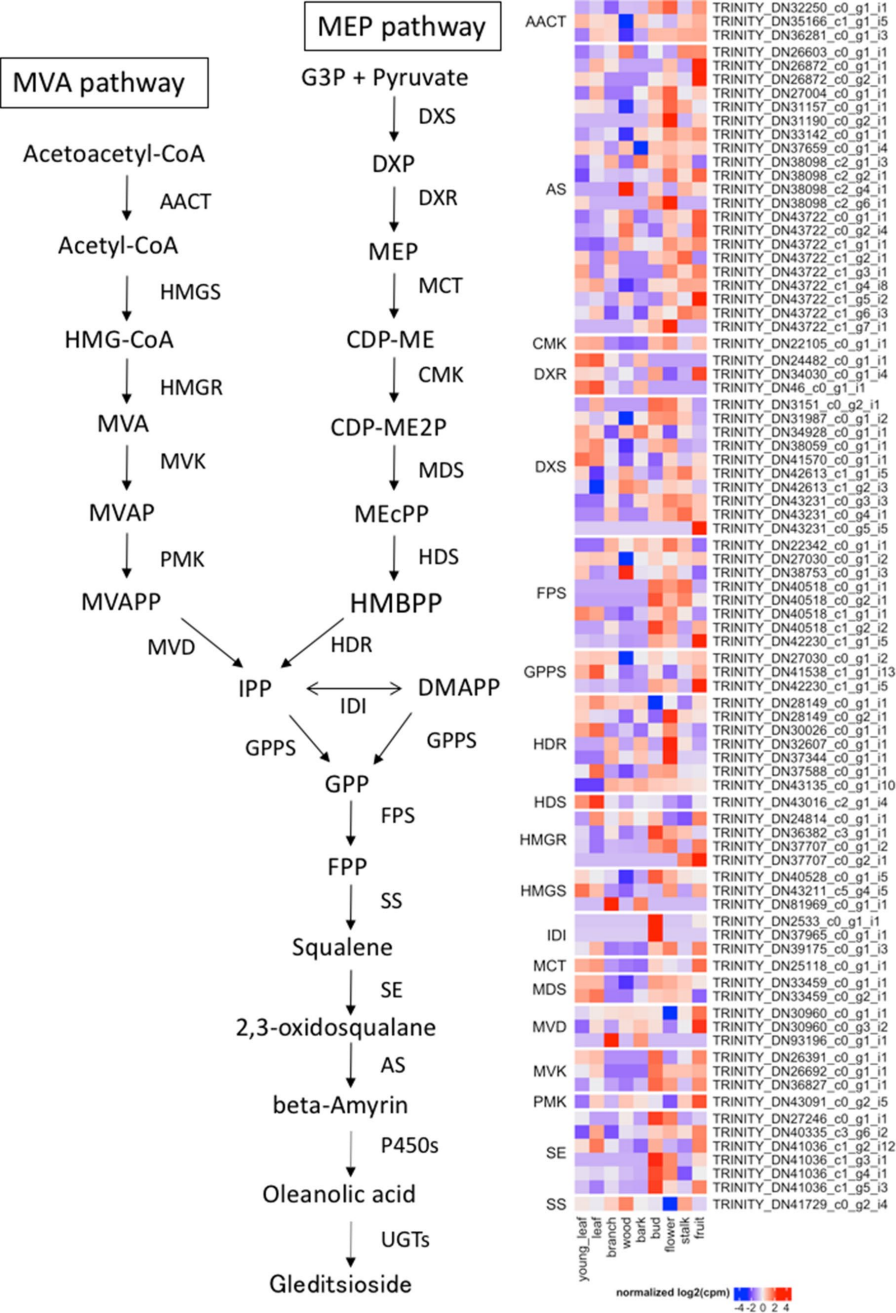
Putative triterpenoid backbone biosynthesis pathway (MVA and MEP) of *G. sinensis* and gene expression profile of the key enzymes

**Fig. 8:**
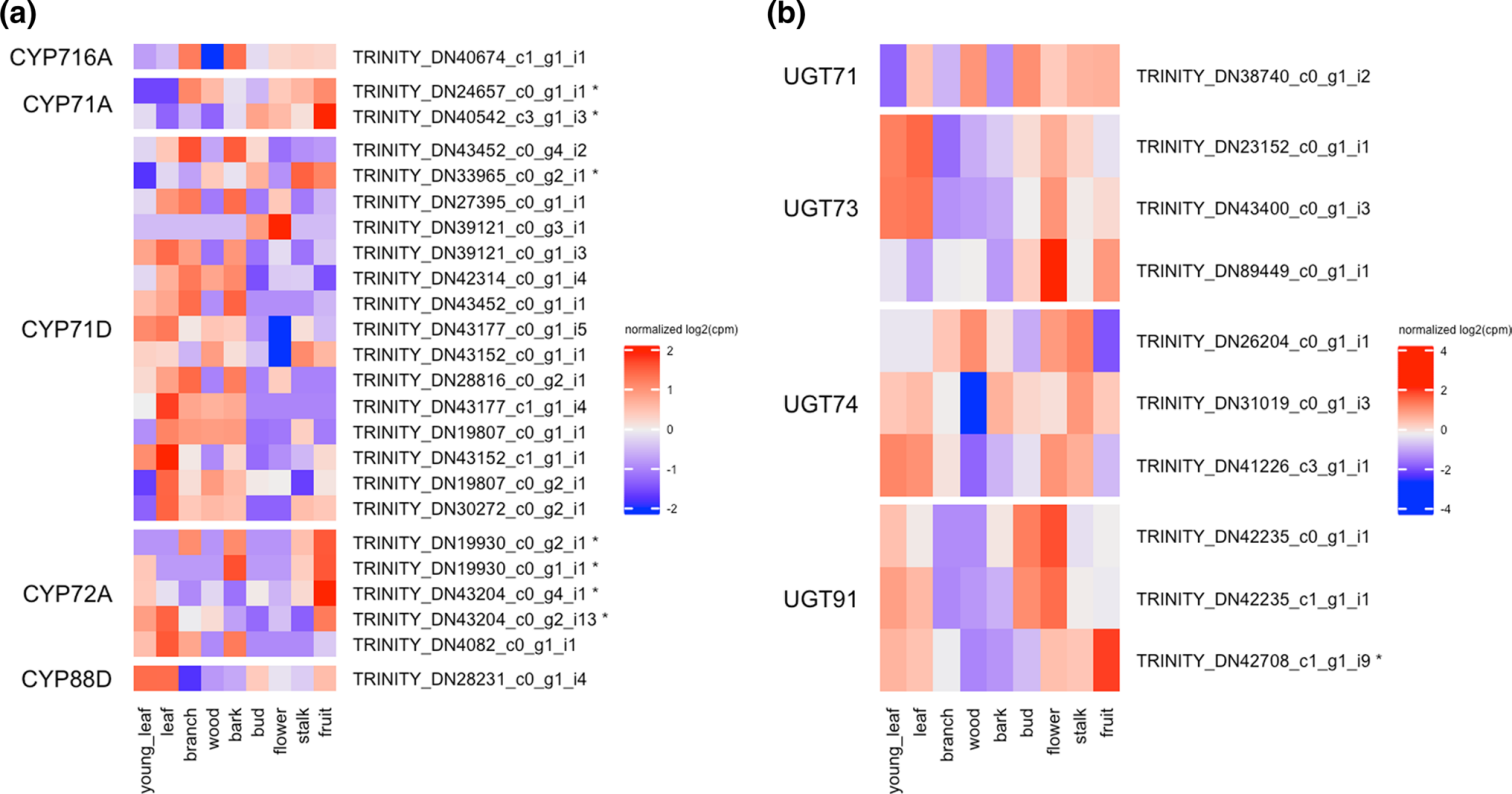
Gene expression profiles of candidate P450s (**a**) and UGTs (**b**) involved in biosynthesis of triterpenoid saponins. Asterisks indicate highly expressed genes in fruit with normalized CPM value more than 1

The mevalonate (MVA) and methylerythritol phosphate (MEP) pathways are essential biosynthetic processes for formation of the triterpenoid backbone (Fig. 7). The heatmap represents the expression profile of putative genes associated with the MVA and MEP pathways (Fig. 7). In the MVA pathway, we annotated three acetyl-CoA acetyltransferases (AACT, EC:2.3.1.9), three HMG-CoA synthases (HMGS, EC:2.3.3.10), four HMG-CoA reductases (HMGR, EC:1.1.1.34), three mevalonate kinases (MVK, EC:2.7.1.36), one phosphomevalonate kinase (PMK, EC:2.7.4.2), and three mevalonate-5-diphosphate decarboxylases (MVD, EC:4.1.1.33). We also annotated within the MEP pathway which MEP is converted to isopentenyl diphosphate (IPP) and dimethylallyl diphosphate (DMAPP), ten 1-deoxy-d-xylulose 5-phosphate synthases (DXS, EC: 2.2.1.7), three 1-deoxy-d-xylulose 5-phosphate reductases (DXR, EC: 1.1.1.267), one 2-*C*-methyl-d-erythritol 4-phosphate cytidylyltransferase (MCT, EC: 2.7.7.60), one 4-(cytidine 5′-diphospho)-2-*C*-methyl-d-erythritol kinase (CMK, EC: 2.7.1.148), two 2-*C*-methyl-d-erythritol 2,4-cyclodiphosphate synthases (MDS, EC: 4.6.1.12), one 4-hydroxy-3-methylbut-2-enyl diphosphate synthase (HDS, EC: 1.17.7.1), and seven 4-hydroxy-3-methylbut-2-enyl diphosphate reductases (HDR, EC: 1.17.1.4). To increase the structural diversity of triterpenoids, triterpenoids were modified with hydroxylation by cytochrome P450 monooxygenases (P450s) and glycosidation by UDP-glycosyltransferases (UTGs) [22]. The P450s form a large family in the plant genome. In recent studies, several P450s were identified as the enzymes involved in saponin biosynthesis. The CYP93E subfamily catalyzes the C24-hydroxylation of beta-amyrin in *Glycine max* [23], the CYP88D subfamily catalyzes the two-step oxidation of beta-amyrin at C11 [24], and the CYP72A subfamily catalyzes the hydroxylation of C22 in 24-hydroxy-beta-amyrin [25]. From our transcriptome data, 136 P450s and 77 UGTs were annotated using HMMER against the Pfam database (Supplementary Table S2). Among these candidates, 26 P450s and 10 UGTs were highly similar to known P450 family members (CYP51, CYP71, CYP716, CYP72, CYP88, and CYP93) and UGT family members (UGT71, UGT74, UGT91, UGT94) involved in saponin biosynthesis. Figure 8a, b show the gene expression patterns of the candidate P450s and UGTs. Our metabolomics analysis and GO enrichment analysis of tissue-specific genes suggested that one of the main tissues of saponin biosynthesis would be fruit. Moreover, we screened annotated unigenes as P450s and UGTs with high levels of expression in fruit. Seven P450s (Fig. 8a) and one UGT (Fig. 8b) that were highly expressed in the fruit of *G. sinensis* were identified as candidate genes involved in the biosynthesis of triterpenoid saponins.

## Discussion

Triterpenoid saponins are major secondary metabolites of *G. sinensis*. Understanding their biosynthesis pathway is important for the high-volume production of cultivated species, but the basic information included in the genome sequencing and transcriptome profile is not sufficient. Advanced sequencing technology helps us to discover new genes involved in secondary metabolite formation and to infer the biosynthesis pathway for medicinal plants. De novo transcriptome studies have been successfully used to infer the saponin biosynthesis pathway in plants such as *Panax japonica*, *Anemone flaccida, Ilex asprella*, and *Platycodon grandiflorus* [26–29].

To our knowledge there has been only one study on the transcriptome analysis of *G. sinensis* [9]. Zhu et al. constructed 142,155 contigs from four tissues based on 77.5 million reads but did not describe saponin biosynthesis. Our de novo transcriptome assembly from the nine tissues yielded 47,855 unigenes with N50 1952 bp based on 143 million reads. The 31,717 high-quality unigenes were annotated with the NCBI-nr protein database using Blast2GO. The annotation revealed that the unigenes were highly similar to those of* Glycine* spp. The ontologies significantly enriched for the genes specifically expressed in fruit were involved in saponin biosynthesis, and the contents of many saponins were high in fruit by the metabolome analysis. These results suggested that saponin biosynthesis is active mainly in the fruit. In fact, oleanane-type triterpenoid saponins, including gleditsiosides I, J, and K, were previously isolated from the fruit of *G. sinensis* [21]. On the basis of the Blast2GO annotation, we identified the 84 genes that may be involved in saponin biosynthesis, including those active in the mevalonate (MVA) and methylerythritol phosphate (MEP) pathways. In the gleditsioside biosynthesis, cyclization of 2,3-oxidosqualene is the first step taken by the enzymes coding oxidosqualene cyclases (OSCs), including beta-amyrin synthase. The cyclic skeletons created by OSCs are oxidized by cytochrome P450 (P450s) to sapogenins, which are the non-sugar part of saponins. The sapogenins undergo glycosylation of the C3-hydroxyl and C28-carboxyl by UDP-dependent glycosyltransferase (UGTs) in order to increase the diversity of saponins. On the plant genomes, 1% of coding genes code P450s, which is the largest family of enzymes. The plant P450 families form a category 11 clan based on their phylogenetic tree [30]. With respect to triterpenoid saponins biosynthesis, the clans CYP51, CYP71, CYP72, and CYP85 have been reported as key enzymes. In the CYP72 clan, DN43204_c0_g4_i1, which has a high similarity to CYP72A219, is specifically expressed in fruit. In *Panax ginseng*, Han et al. reported that CYP72A219 encodes secologanin synthase, which catalyzes the conversion of loganin to secologanin. In the CYP85 clan, DN28231_c0_g1_i4 and DN40674_c1_g1_i1 are highly similar to CYP88D3 and beta-amyrin 28-oxidase, respectively. The function of CYP88D3 is not clear, but Seki et al. identified CYP88D6 as a beta-amyrin 11-oxidase in *Glycyrrhiza* plants [24]. In addition, DN42708_c1_g1_i9 was similar to UGT91A1, which was predicted to be involved in the glycosylation of flavonols or flavonol glycosides in *Arabidopsis thaliana* [31]. The function of these candidate P450s and UGTs involved in triterpenoid saponin biosynthesis has not been well characterized in *G. sinensis*, but de novo transcriptome analysis could help us to identify important candidates for future functional characterization.

## Conclusions

In this study, we performed de novo transcriptome analysis and metabolome analysis using nine tissues of *G. sinensis*. Our transcriptome data were more comprehensive than the data in previous reports. This data set will help to elucidate the secondary metabolite biosynthesis pathway for the breeding of medicinal plants with a high yield of saponin, and thereby accelerate the future commercial production of saponins.

## Electronic supplementary material

Below is the link to the electronic supplementary material.

Supplementary material 1 (TIFF 26370 KB)

Supplementary material 2 (XLSX 32 KB)

Supplementary material 3 (XLSX 2610 KB)

Supplementary material 4 (XLSX 59 KB)
