# Supplementary figures and images for: Identification of potential genes involved in triterpenoid saponins biosynthesis in Gleditsia sinensis by transcriptome and metabolome analyses
Source: J Nat Med. 2018 Dec 13;73(2):369–80. doi: 10.1007/s11418-018-1270-2 (PMC6373339; doi:10.1007/s11418-018-1270-2)

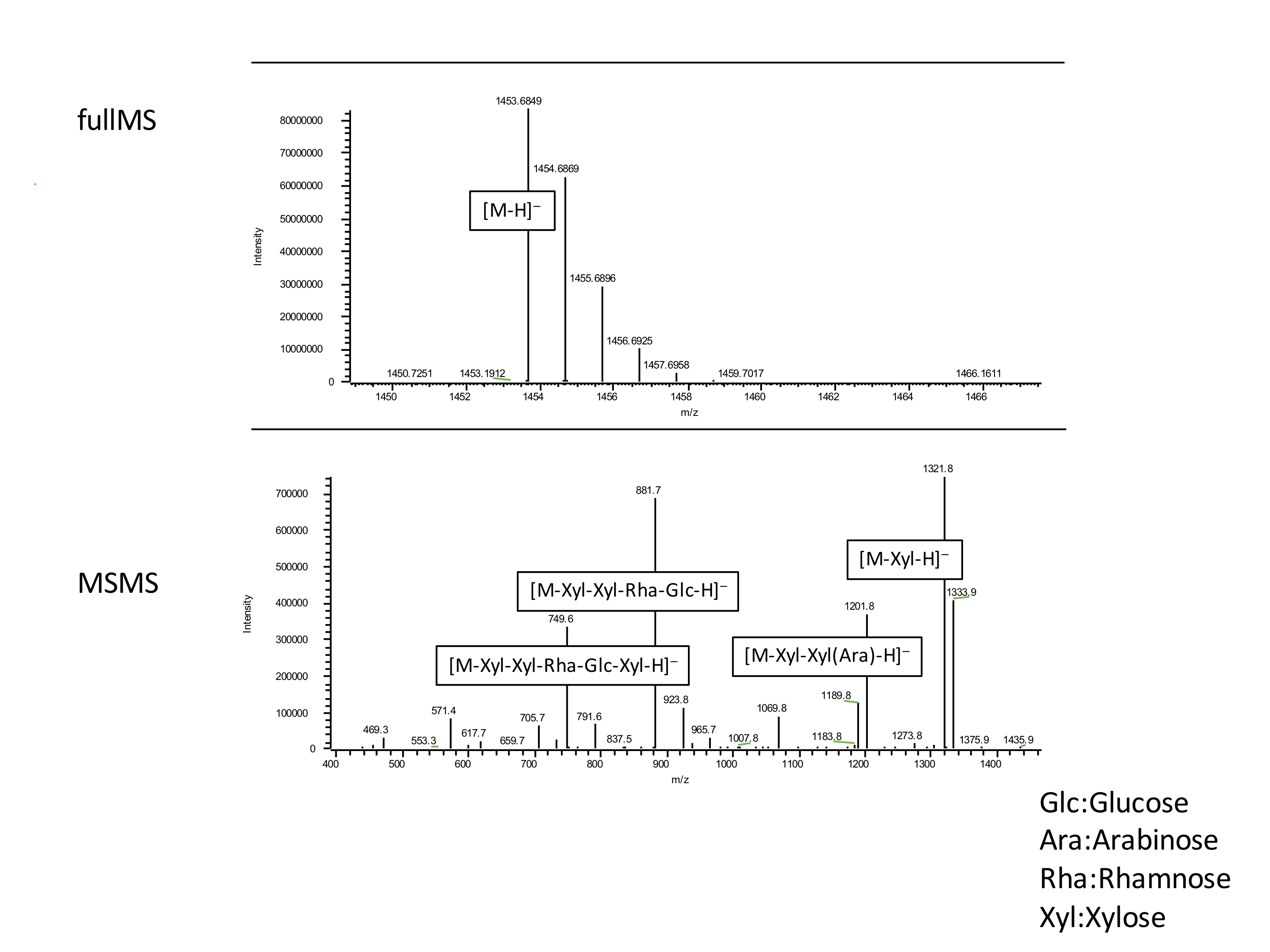

Supplement: Supplementary file 1 — Supplementary material 1 (TIFF 26370 KB) [file 11418_2018_1270_MOESM1_ESM.tiff]
